# Prevalence of mental diseases in Austria: Systematic review of the published evidence

**DOI:** 10.1007/s00508-018-1316-1

**Published:** 2018-01-24

**Authors:** Agata Łaszewska, August Österle, Johannes Wancata, Judit Simon

**Affiliations:** 1 Department of Health Economics, Center for Public Health, Medical University of Vienna, Kinderspitalgasse 15/I, 1090 Vienna, Austria; 2 Institute for Social Policy, Vienna University of Economics and Business, Welthandelsplatz 1, 1020 Vienna, Austria; 3 Clinical Division of Social Psychiatry, Department of Psychiatry and Psychotherapy, Medical University of Vienna, Währinger Gürtel 18–20, 1090 Vienna, Austria

**Keywords:** Mental disorders, Austria, Prevalence, Systematic review, Survey methods

## Abstract

**Background:**

Addressing the growing burden of mental diseases is a public health priority. Nevertheless, many countries lack reliable estimates of the proportion of the population affected, which are crucial for health and social policy planning. This study aimed to collect existing evidence on the prevalence of mental diseases in Austria.

**Methods:**

A systematic review was conducted using MeSH, EMTREE and free-text terms in seven bibliographic databases. In addition, the references of included papers and relevant Austria-specific websites were searched. Articles published after 1996 pertaining to the Austrian adult population and presenting prevalence data for mental diseases were included in the analysis.

**Results:**

A total of 2612 records were identified in the database search, 19 of which were included in the analysis, 13 were community-based studies and 6 examined institutionalized populations. Sample sizes ranged from 200 to 15,474. The evidence was centered around depression (*n* = 6, 32%), eating disorders (*n* = 4, 21%) and alcohol dependence (*n* = 3, 16%). While most studies (*n* = 10, 53%) used questionnaires and scales to identify mental diseases, seven studies used structured clinical interviews, and two studies examined use of psychotropic drugs. Due to the diversity of methodologies, no statistical pooling of prevalence estimates was possible.

**Conclusion:**

Information on the prevalence of mental diseases in Austria is limited and comparability between studies is restricted. A variety of diagnostic instruments, targeted populations and investigated diseases contribute to discrepancies in the prevalence rates. A systematic, large-scale study on the prevalence of mental diseases in Austria is needed for comprehensive and robust epidemiological evidence.

**Electronic supplementary material:**

The online version of this article (10.1007/s00508-018-1316-1) contains supplementary material, which is available to authorized users.

## Introduction

Mental diseases are associated with high economic and societal burden and reduction in the quality of life of those affected [1, 2]. According to the Global Burden of Disease Study 2010, the burden of mental diseases has increased by 38% worldwide between 1990 and 2010 and is continuously growing [3]. On average around 38% of the population of the European Union are affected each year by mental health problems [4]. Among the European countries, the lifetime prevalence was estimated at 41% and 43%, whereas 12-month prevalence was 23% and 31% in the Netherlands and Germany, respectively [5, 6]. Evidence from a more recent study, the European Study of the Epidemiology of Mental Disorders (ESEMeD), showed that nearly 1 in 10 respondents reported the presence of affective disorders, anxiety disorders and/or alcohol abuse (not including other somatoform disorders or substance abuse) in the past 12 months [7]. A recent meta-analysis showed that the mortality rate among people suffering from mental diseases is more than two times higher compared to the general population or people without mental diseases [8].

An accurate account of the proportion of the population affected by mental diseases in many countries is still lacking [9] hindering opportunities for detection of unmet needs, prevention and early interventions. International differences in populations and healthcare systems also highlight the importance of country-specific epidemiological studies [10]. Comparisons between existing evidence, however, are impeded by the heterogeneity of choice of concepts and methods used to estimate the prevalence rates of mental diseases [1, 11–13]. These are mostly related to the use of different case-finding tools, i. e. the instruments utilized for identifying psychiatric cases [6, 14]. Common methods of case-finding include the use of either structured clinical interviews, e. g. Schedules for Clinical Assessment in Neuropsychiatry (SCAN), which allow classification of diseases to categories of the Diagnostic and Statistical Manual of Mental Disorders (DSM) and International Classification of Diseases (ICD) [15], or screening instruments, mostly questionnaires and symptom rating scales that are able to assess psychological symptoms of diseases rather than clinical diagnoses [16]. Both methods have been used across epidemiological studies [13]; however, it has been shown that screening instruments include numerous false positive results and therefore report generally higher prevalence estimates in comparison to the studies that used DSM or ICD diagnostic criteria [13]. Structured clinical interviews are more able to separate symptoms among specific mental diseases or from those linked to physical illnesses [17]. They are, however, very resource intensive as they require a clinician or researcher trained in psychiatry/psychology to conduct interviews [18], whereas symptom scales are mostly self-reported questionnaires completed by a study participant with or without the assistance of a researcher.

Austria, similarly to other high-income countries, has been experiencing changes in the provision of mental health services since the 1970s [19]. These changes were based on the idea of reorganizing provision of mental health care from large, centralized institutions to decentralized community-based care [20]. While the number of mental health hospital beds was reduced by 62% between 1970 and 2002 [21], the length of stay for mental diseases shortened from 38.3 to 16 days between 1990 and 2000 (58%) and has remained on the same level since (15.9 days in 2014) [22].

Although the structural changes in mental health care are still ongoing, these are not based on relevant evidence for needs. With no access to uniform epidemiological data, such as specialist registries, comprehensive estimates on the population mental health are currently only available from administrative data from the 19 healthcare funds in Austria. According to these, around 900,000 people (approximately 10% of the general population [23]) received mental health care in 2009 [24]. The frequency of mental health conditions as causes for disability pensions have tripled (10.8% in 1995, 35.6% in 2015) [25, 26]. In 2015, 2.3% of sick leave cases, 9.2% of all sick days, and the second highest average length of a sick leave at 38 days were attributed to mental diseases [27]; however, these estimates are likely to be great underestimations due to the substantial degree of unmet needs for treatment, the misdiagnoses, stigma and coding bias similarly to other EU countries [1]. For example, Alonso et al. (2004) found that approximately two thirds (63%) of people suffering from depression did not receive any treatment in the last 12 months [28].

Robust epidemiological estimates on the occurrence of a disease are central to adequate health care planning, and prevention and promotion activities. Systematic reviews are important tools to provide this information in an integrated form [29]. In Austria, information available in the peer-reviewed literature on the epidemiology of mental disorders has not yet been collated and presented in a systematic and aggregated form. The first objective of this study was to collect and synthesise available information on the prevalence of mental diseases among the adult Austrian population. The second objective was to describe the comparability of the different applied methods, including case-finding.

## Methods

### Database search

A systematic literature review was carried out to collect all evidence on the epidemiology, i. e. prevalence, incidence and mortality, of mental diseases among the adult population in Austria published between January 1996 and May 2016. Searches were conducted in seven bibliographic databases: MEDLINE, EMBASE, Scopus, PsycINFO, CINAHL, Social Science Citation Index (SSCI) and PSYNDEXplus. The literature search used a combination of MeSH, EMTREE and free-text terms (Supplementary material Table 1). Furthermore, searches for relevant literature included inspecting the reference lists of included articles and searching national websites (i. e. Statistik Austria, LBI HTA, Gesundheit Österreich GmbH, Hauptverband der österreichischen Sozialversicherungsträger and HIS) using German search terms. The searches were performed in May 2016.

### Screening and eligibility

First, screening of titles and abstracts was performed to exclude non-relevant articles. Full-text screening of potentially relevant papers was then carried out. The following exclusion criteria were applied: (1) it was not possible to extract epidemiological data for the Austrian adult population, (2) studies and reports referring only to statistics on hospital admissions and discharge rates, (3) duplicate articles or multiple publications of the same study population, (4) only abstracts or conference papers, and (5) studies published before 1996. Initially no language filter was applied but ultimately, studies in English and German were included.

Mental disorders of interest included: psychotic disorders (schizophrenia, schizoaffective disorders), mood disorders (depression, bipolar disorder, dysthymia), anxiety disorders, stress-related disorders, eating disorders and substance use disorders. Neurodevelopmental disorders (e. g. intellectual disabilities, autism, attention-deficit disorders), neurocognitive disorders (e. g. dementia) as well as mental diseases described as comorbidities of other health problems were not considered. If multiple publications for the same study population presenting the same epidemiological data were found, the most informative (i. e. allowing extraction of more data for the analysis) article was considered.

In this review, the term “epidemiology” is defined as any data on prevalence, incidence or mortality related to mental diseases. “Prevalence” is defined as the proportion of population that suffers from a defined condition at a defined point or period of time, e. g. 12-month prevalence, lifetime prevalence and “incidence” as the number of new occurrences of a health problem in a defined period of time [30].

### Study quality

The quality of the studies was assessed using the criteria for critical appraisal of prevalence and incidence studies developed by Loney et al. (1998) [31]. This consists of a scoring system that rates eight quality criteria. A score of 1 or 0 is assigned to each item making 8 the maximum score that could be obtained [31]. No cut-off point was suggested by the authors.

### Analysis

Similar to other relevant international reviews [32, 33], this article includes studies based both on general and institutionalized populations (i. e. nursing homes, hospitals, prisons). Findings are summarized and presented separately. Due to the diversity of methodologies, settings and study outcomes among the included publications, no statistical pooling was possible. Therefore, main findings were synthesized narratively.

## Results

**Fig. 1:**
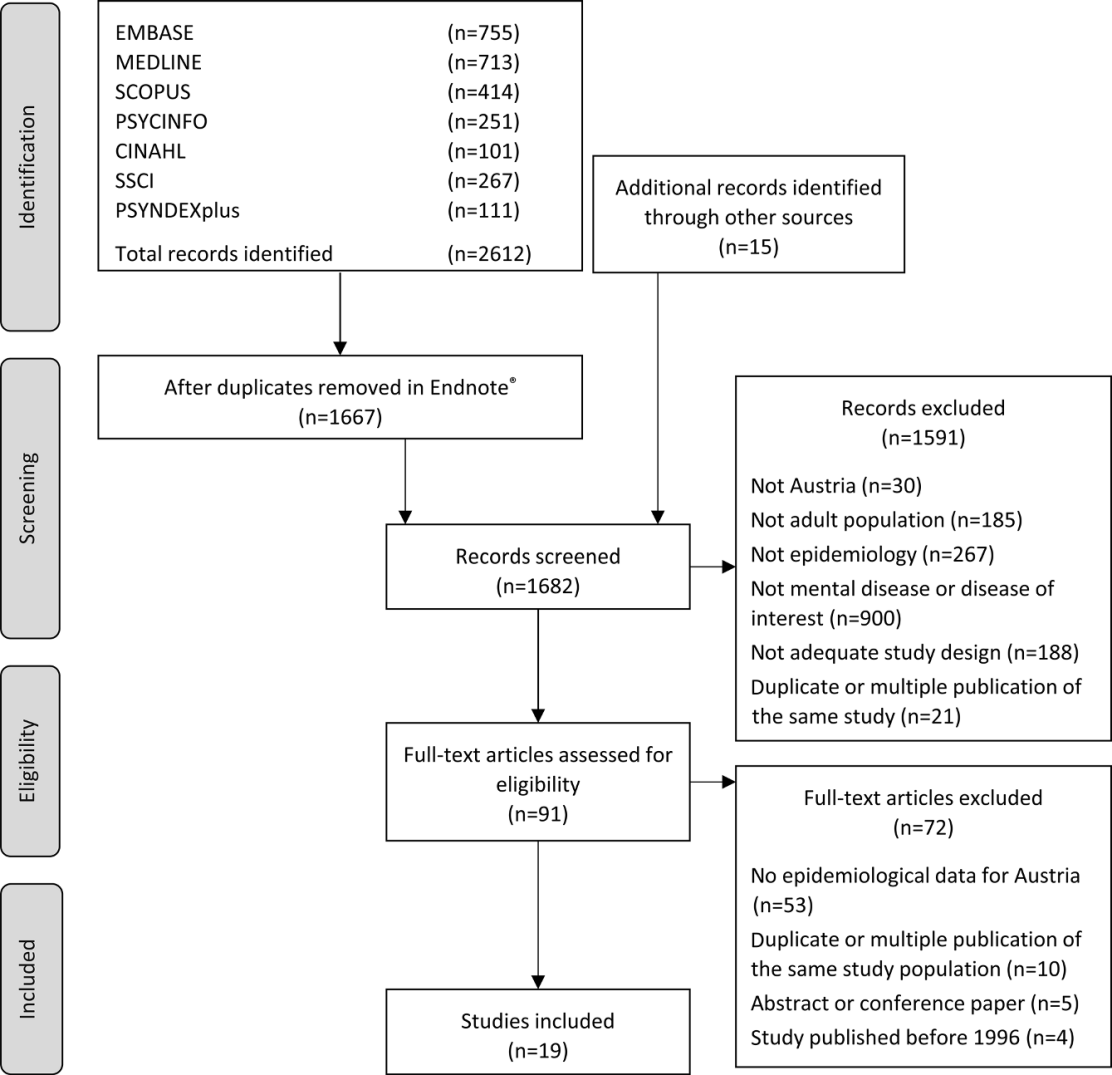
Flow diagram of the systematic literature search. *Endnote X7 was used for reference management

A total of 2612 records were identified in the databases search and 15 reports through the website search. First and second-level screening resulted in a total of 19 peer-reviewed articles on the epidemiology of mental diseases in Austria which were included in the analysis. None of the reports from the website search were suitable for inclusion as no epidemiological data meeting the inclusion criteria were available. A flow diagram of the literature search based on the Preferred Reporting Items for Systematic Reviews and Meta-Analyses (PRISMA) [34] is presented in Fig. 1. A detailed description of the included articles is presented in Supplementary Material Table 2.

More than a half of the articles were community-based studies with randomly selected samples (*n* = 12) [35–46] and one examined a sample of 18-year-old men assessed for a mandatory military service in one Austrian state [47]. Out of the remaining six studies, three were conducted among patients of general hospital wards [48–50], three among nursing home residents [50–52] (one study included both nursing homes and hospitals [50]), and one examined a sample of prison inmates of one prison located in Vienna, Justizanstalt Wien-Josefstadt [53]. Sample sizes ranged from 200 to 15,474 individuals. The year of identified prevalence estimates varied from 1991 to 2011. The majority of studies were national studies (*n* = 15) and four articles pertained to international studies where two or more counties were participating [35, 39, 44, 50]. Different methods of acquiring data were used. Out of the 12 studies that were based on randomly selected samples, three used data from existing international surveys (European Health Interview Survey, European Social Survey Round 3 and Survey of Health, Ageing and Retirement in Europe) [35, 39, 44]. The remaining nine used their own sampling methods which included using telephone directories [36, 42, 43], census bureau data [37, 38, 40, 54] and district voting registries [41, 45]. Of the studies six selected study participants from the population residing in institutions [48–53] and one study assessed a sample of 18-year-old men assessed for a mandatory military service in one Austrian state [47]. Out of all articles included (*n* = 19), diagnostic interviews were applied in six studies [41, 45, 48, 49, 52, 53]. Otherwise, evidence was collected using self-completed questionnaires (*n* = 7) [35, 39, 40, 43, 44, 46, 47], telephone surveys (*n* = 4) [36–38, 42], or obtained from patient medical records [51]. In one study both diagnostic interviews and self-completed questionnaires were used [50].

Most studies presented results for point (current) prevalence of mental diseases (*n* = 15) [35, 37–40, 43, 45, 47–54], others reported 10-year [42], 2‑week [36] and 1‑week [44] prevalence rates. One study reported incidence [41].

The quality scores of the studies ranged from 3 to 6 with a mean of 4.79 (SD 1.03), using criteria adopted from Loney et al. (1998) [31]. Of the studies 10 used trained interviewers to minimize a possible measurement bias [35, 37–39, 41, 45, 48, 49, 51, 52]. Study subjects were randomly selected from the general population in 10 studies thus minimizing the potential for selection bias [35–40, 42–44, 46]. A summary of prevalence estimates found in the review is presented in Supplementary material Table 3 for the general population and Table 4 for institutionalized populations. A short summary of the main findings is provided.

### Mental diseases in the general population

A total of 13 community-based studies were included. None of the studies examined the overall prevalence of all psychiatric disorders within the general Austrian population. The primary outcome was to identify the prevalence of depressive disorders (*n* = 6) [35, 36, 41, 42, 44, 55], followed by eating disorders (*n* = 4) [37, 38, 40, 46], two studies examined the prevalence of alcohol dependence [43, 47] and one study looked at individuals who received psychopharmacological treatment [39].

#### Depression, seasonal depressive disorder and anxiety

Of the studies six examined the prevalence rates of depression in the general population [35, 36, 41, 42, 44, 45]. The sample size varied from 331 to 3448 and the prevalence estimates ranged from 9.9% (2-week prevalence) to 15.6% (10-year prevalence) for the adult population and from 16% to 19.6% (point prevalence) for older adults.

The 10-year prevalence of seasonal depressive disorder (SAD) was 2.5% and 1.8% applying DSM-V and ICD-10 criteria, respectively [42]. In addition, one study found the incidence of new cases of depression in the respondents aged 75 years and above with no history of depression at 16.6% over 30-month follow-up [41].

#### Eating disorders

A total of four studies examined the epidemiology of eating disorders in the Austrian population [37, 38, 40, 46] with sample sizes varying between and 475 and 1000 respondents. Among women aged 40–60 years, point prevalence of eating disorders defined as occurrence of anorexia nervosa, bulimia nervosa or eating disorders not otherwise specified (EDNOS) was estimated at 4.6%. In addition, 4.8% of women met the criteria for subthreshold eating disorders [46]. Slightly lower point prevalence rates were observed in women within the age group 60–70 years; 3.8% had eating disorders and 4.4% had single symptoms of eating disorders [40]. In another sample of women aged 18–85 years, 3.3% were diagnosed with binge eating disorder (BED) and 1.5% suffered from bulimia nervosa [38]. Among men, the point prevalence of BED, partial BED and EDNOS was found to be 0.8%, 4.20%, and 0.50%, respectively. The total prevalence of clinical and subclinical eating disorders was surprisingly high for men (14.9%) [37]; however, it is difficult to interpret this high rate as the authors did not provide a definition of subclinical eating disorders.

#### Alcohol and substance dependence

Rathner et al. (1998) examined the prevalence of alcohol dependence across the Austrian population. The study was conducted in 1996 and showed that 2.2% of the studied population at the time of the study were alcohol dependent [43]. In another study, among all 18-year-old males undergoing assessment for National Military Service in Lower Austria, 3.2% were identified as having alcohol dependence, whereas 15% had issues with alcohol abuse [47].

#### Psychotropic treatment

Kopp et al. (2011) reported based on the Austrian Health Interview Survey (2006/2007) that out of 15,474 interviewed adults, 5.7% had received psychopharmacological treatment in the last 12 months for depression, anxiety or both [39].

### Mental diseases in the institutionalized population

A total of six articles examined mental diseases in institutionalized populations. Of these, three articles referred to nursing home residents [50–52], three were concerned with patients of general (non-psychiatric) hospital wards [48–50] (one study included both nuring homes and hospitals [50]) and one was conducted in a prison [53].

#### Nursing homes

At least one psychiatric or neurological disorder was diagnosed in 76% of the 262 newly admitted nursing home residents in Tyrol and Vienna in 1991–1992 [52]. The most common diagnoses fell into the categories dementia or other organic illnesses (64.9%), adjustment, neurotic or psychosomatic disorders (16.8%), substance abuse disorders (4.2%), depressive disorders (3.4%), manic depressive or other psychoses (2.3%) and schizophrenia (1.9%) [52]. Another study showed that roughly 75% of the residents of nursing homes in the Austrian state of Vorarlberg received psychotropic medication at least once in the preceding year (36.8% of them received antidepressants) [51].

Assessed with the Structured Clinical Interview for DSM-IV (SCID), alcohol dependence was diagnosed in 0.7% of nursing home residents in a combined sample of 33 nursing homes from the Austrian state of Salzburg. Problematic alcohol consumption was identified in 5.7% and 5.5% of study participants using the Alcohol Use Disorder Identification Test (AUDIT) and the Short Michigan Alcoholism Screening Test (SMAST-G), respectively [50]. In addition, 2.6% of the nursing home residents were diagnosed with abuse of or addiction to medication [50].

#### General hospital wards

Approximately one third (32.2%) of the 821 patients of general (non-psychiatric) hospital wards were diagnosed with mental diseases [48]. Dementia was the most frequent (11.6%), followed by depression (10%) and substance abuse disorders (5.8%) [48]. A similar study estimated the prevalence of psychiatric disorders at 30% among 784 patients of the general hospital wards [49].

#### Prisons

Of the studies one examined the prevalence of psychiatric disorders among both convicted and remand prisoners in one prison located in Vienna [53]. A high proportion of the studied sample was diagnosed with one or more mental disorders (72% convicted and 69% remanded in custody prisoners). Particularly, substance dependence and substance use were common among prisoners as over half of the prisoners (54% and 57% for convicted and remanded in custody prisoners, respectively) regularly consumed one or more substances. Mood disorders were found in 4% of the convicted prisoners and 13% of the remand prisoners. Schizophrenia was diagnosed in 3% of both groups [53].

### Differences according to gender and socioeconomic status

Only six studies mentioned statistically tested differences in the prevalence rates between men and women, three studies reported that there was no statistically significant difference in the occurrence of major depression or SAD [42], or depression [36] and mental diseases in general [49]. In a fourth study, the occurrence of anxiety disorder was found to be significantly higher for women [36] and two further studies reported that men suffer more often from alcohol dependence [43, 50]. In terms of socioeconomic status, women with a lower education level and non-European origin were more likely to suffer from eating disorders [46]. Moreover, migrant women from Eastern Europe had significantly higher prevalence estimates both for depression and anxiety compared to women with an Austrian origin [36].

Study respondents who received psychopharmacological treatment were more likely to be older, female, living alone and have lower income [39]. Meanwhile, being single and female were both associated with significantly higher benzodiazepine use among older people (75+ years) [55].

## Discussion

The aims of this literature review were to collate, summarize and compare existing published evidence on the prevalence of mental diseases in Austria and to highlight information gaps.

### Summary of the main findings and international comparison

Based on the findings of this review we can conclude that between 9% (point prevalence) and 15% (10-year prevalence) of the adult population in Austria may be affected by depression, 6.5% by anxiety disorders, and 2.5% by SAD. Slightly higher prevalence rates for depressive disorders have been observed in older adults (50+ years) and older persons (75+ years, 19.6% and 16.3%, respectively). The prevalence of eating disorders was surprisingly high in the Austrian population. Nearly 5% of women aged ≥40 years had symptoms of eating disorders. Around 2% of the general population and 3.2% of 18-year-old male Austrians had problems with alcohol dependence. Nearly 6% of the individuals in a household survey reported receiving psychopharmacological treatment. Over 30% of patients of non-psychiatric hospital wards were suffering from mental health conditions and more than 70% of institutionalized individuals (nursing homes and prisons) were diagnosed with a mental disease.

Comparisons between the evidence from Austria and international literature should consider the variety of diagnostic methods applied as well as variations between the studied groups [11]. Characteristics of the study samples in terms of different age groups as well as different definitions of mental diseases adopted may lead to varying results [6, 14].

The point prevalence rates of major depression in Europe reported by Wittchen et al. were reported at 6.9% in 2011 [4] and 8.3% in 2005 [1] and were lower than the estimates reported for Austria (9% [36]). In comparison to the global evidence, the point prevalence of major depression was also higher in Austria (9% vs. 4.7%) [13]. The average lifetime prevalence estimates of major depression in 10 high-income countries (14.6%) reported by Bromet et al. (2011) [56] was close to the 10-year prevalence reported by Pjrek et al. (2016) for Austria (15.60%) [42].

The estimate for major depression (MD) in the elderly population in Austria (point prevalence of 7.2% for population 75+ years [45]) was also higher comparing to that reported by Volkert et al. (2013), who found the prevalence of MD in older people in Europe and North America at 3.29% for current and 16.52% for lifetime MD [57].

Surprisingly, only six studies reported differences in the prevalence rates between men and women of which three studies reported that there were no statistically significant differences in the prevalence rates of depression [36, 42], SAD [42] and mental diseases in general [49]. This is in contrast to international published evidence showing that women have about twice as high risk of suffering from depression [13, 56] or non-seasonal depression [58] than men.

No studies on mortality and only one study on incidence was found in the review. This is not surprising as incidence estimates require clinical studies with long periods of follow-up and such studies are therefore more costly to conduct and more prone to loss to follow-up than prevalence studies. Estimating mental diseases-related mortality has additional methodological difficulties due to frequent comorbidities. According to the international literature, lower socioeconomic status is associated with poorer mental health [59] and higher costs of healthcare services [60, 61]. Future policies should also consider looking at the value of different treatment options in the light of important and potentially amenable socioeconomic factors associated with mental diseases. To be able to indicate key areas for healthcare service planning, these economic factors should be researched along with the epidemiological data. This review could identify only marginal evidence on these associations.

### Comparability of the applied methods

Different approaches to case-finding were utilized in the various study samples. Even among the relatively small number of studies included in this review, a variety of different case-finding instruments was observed. For example, among six studies presenting data on depression, seven different instruments were used and four different measures were used to assess alcohol dependence in the three relevant identified studies. These results highlight the lack of standardized methods.

In general, five studies derived prevalence estimates based on cut-off values on a self-reported questionnaire index which may have led to misinterpretation. Respondents who scored high on a self-reported scale had symptoms of mental diseases but were not classified as exhibiting a clinical condition. This limitation of screening questionnaires was mentioned by multiple authors using the Centre for Epidemiologic Studies Depression (CES-D) scale [35, 44]. On the other hand, some screening questionnaires may result in considerable amount of false positive cases and overestimate the prevalence rates [16, 62]. This aspect has been well demonstrated in one study examining alcohol dependence among nursing home residents. While the occurrence of alcohol dependence was 0.7% using a structured clinical interview, the prevalence rates measured by screening instruments were higher, 5.7% and 5.5% using AUDIT and SMAST-G, respectively [50].

The use of different cut-off values on some symptom scales may cause other problems with comparability between studies. For instance, Kapusta et al. (2006) used a cut-off value of ≥2 on the CAGE alcohol screening questionnaire to diagnose alcohol dependence [47], whereas Rathner and Dunkel (1998) used a cut-off value of 4 [43]. If the approach of ≥2 points on the CAGE scale would be adopted in Rathner and Dunkel (1998), 21% of the respondents would fall into a category of alcohol dependence, compared to the 3.2% reported (for 18-year-old men) by Kapusta et al. (2006). The circumstances of collecting data, i. e. mailed questionnaire vs. completing a questionnaire during the examination for mandatory military service, could also contribute to differing results. While Bongers and Van Oers (1998) did not observe differences in self-reported alcohol use assessed by mail survey and personal interviews [63], more recently Dotinga et al. (2005) reported that higher alcohol use in a mailed survey in comparison to face-to-face interviews [64]. Mangweth-Matzek et al. [46] and Von Dem Knesebeck et al. [44] also used two different cut-off values on the CES-D scale in their studies to assess depressive symptomatology among the studied samples. Even though the studies were concerned with the same mental health problem, a direct comparison of prevalence rates was, therefore, not possible.

Another important factor hindering comparisons between studies is the type of the prevalence estimate reported, e. g. point prevalence, lifetime prevalence. While the 10-year prevalence of depression was reported at 15.6% [42], a 2‑week prevalence was estimated at 9.9% [36]. Both estimates were for the general Austrian population but measured with different screening instruments.

Finally, data collection methods and sample self-selection could further contribute to variations in the findings and their generalizability. One study based the prevalence rates of eating disorders on mail questionnaire responses from a sample selected based on the census bureau data. As the response rate did not reach 50%, the generalizability of findings is considered limited [40, 46]. In four other publications the data presented were obtained via telephone interviews [36–38, 42]. Relevant potential limitations to consider are: language barriers and lower response rate among migrant or lower socioeconomic groups as reported for example by Kerkenaar et al. (2013) [36].

### Limitations of this review

The results of this review should be interpreted in the light of certain limitations. Firstly, neurodevelopmental and neurocognitive disorders were not considered in the analysis. This might limit comparisons with the international literature where neurological and mental diseases were analyzed together (e. g. Global Burden of Disease Study [3], cost of disorders of the brain in Austria [65], Wittchen et al. (2011) [4]). Secondly, the paper focuses on studies investigating mental diseases as primary diagnoses, and studies describing mental diseases as comorbidities or consequences of other health problems were excluded. This leads to a systematic underestimation of the real scope of mental health problems in Austria. Thirdly, some relevant information may be missed, if an article did not explicitly refer to mental diseases in its title, abstract or keywords (e. g. [66]). At the same time, a comprehensive check of the references of the included articles and a search of the relevant websites should have ensured identification of the most relevant direct evidence. Furthermore, studies and reports containing information only on hospital admissions or discharge rates were excluded as many experts argue that these data do not represent accurate estimates due to coding bias and an underestimation of the real prevalence rates [67]. According to the data from the national health insurance funds in Austria, 0.8% of insured individuals with a primary psychiatric diagnosis were hospitalized in 2007 which constituted approximately 5% of all hospitalizations [68].

### Considerations for future research

Institutionalized populations, i. e. residents of long-term care homes, prisons and hospitals, are often excluded from population-based epidemiological and health services studies [7, 67]; however, the high occurrence of mental diseases among these groups indicates that this is a major limitation in the current evidence-base [52]. The review showed that more than 70% of Austrian nursing home residents and prisoners are potentially affected by mental and neurological diseases. These results are in line with the international literature on the prevalence of mental health problems in long-term care homes [69] and studies on the incarcerated population in the USA and Europe [70].

The problem of general under-recognition and undertreatment of mental illnesses was also highlighted in a few studies [45, 48, 55]. A study on the older Viennese population showed that depression in this group is immensely undertreated. Over half of the study participants diagnosed with major and minor depression did not receive psychotherapeutic or pharmacological treatment [45, 55]. These findings are in line with both national and international evidence. For example, Topitz et al. (2015) reported that only 46% of the patients with depression were recognized by ward physicians as being mentally ill and merely 21% of patients suffering from depression had a documented mental health disease diagnosis at discharge [71]. It is unclear, however, whether concerns over stigmatization of the patients or the insecurity of the doctors regarding diagnoses caused this behavior [71]. Kessler et al. (1994) reported that among respondents suffering from at least one mental health problem during their lifetime, less than half received any professional healthcare and only one fifth received care in the mental healthcare sector [72]. Findings from other international studies arrived at similar conclusions. Lecrubier (2007) also found that only around half of the patients diagnosed with depression received any treatment [73]. These results confirm a great unmet need for interventions in patients with mental illnesses and the possibility that a lack of diagnosis could be a contributing factor to the lack of care received.

Internationally, specific epidemiological studies were conducted to outline the scope of mental diseases in a respective country or multiple European countries. Such studies were conducted for example in Germany (German Health Interview and Examination Survey, GSH) [6], USA (National Comorbidity Survey, NCS) [72], UK (National Psychiatric Morbidity Surveys of Great Britain, NPMS) [15], the Netherlands (The Netherlands Mental Health Survey and Incidence Study, NEMESIS) [5] and in Europe (European Study of the Epidemiology of Mental Disorders, ESEMED) [7]. These studies provide the most reliable estimates of mental diseases in general populations and can serve as examples for future research. For Austria, no similar study has been published so far, neither in scope (the number of people) nor in detail (the types of disorders).

## Conclusion

Currently, due to the lack of robust epidemiological evidence, decisions on mental healthcare services planning are based on fragmented administrative and historical data in Austria [74]. So far, no large representative population-based study has been published in Austria that would cover a comprehensive range of psychiatric diagnoses together with relevant socioeconomic and demographic information. Due to the variety of methods and diagnostic tools used, the non-representative samples, high drop-out rates and other limitations, existing evidence on mental diseases prevalence is not comparable, generalizable or robust enough for planning purposes. The information consolidated in this paper highlights the need for more detailed, representative studies using validated methods and measures that would provide a better understanding of the scope of different types of mental diseases in the Austrian population.

## Caption Electronic Supplementary Material

Design of the search strategy in electronic databases (Table 1), detailed description of the included articles (Table 2), prevalence rates for mental diseases extracted from the included studies (Table 3 and Table 4).
