# Supplementary material for: Prevalence of mental diseases in Austria: Systematic review of the published evidence
Source: Wien Klin Wochenschr. 2018 Jan 24;130(3):141–50. doi: 10.1007/s00508-018-1316-1 (PMC5816100; doi:10.1007/s00508-018-1316-1)
Supplement: Supplementary file 1 — Design of the search strategy in electronic databases (Table 1), detailed description of the included articles (Table 2), prevalence rates for mental diseases extracted from the included studies (Table 3 and Table 4). [file 508_2018_1316_MOESM1_ESM.docx]

**Electronic Supplementary Material**

**Prevalence of mental diseases in Austria: systematic review of the published evidence**

Journal: Wiener klinische Wochenschrift

A. Łaszewska^1*^, A. Österle^2^, J. Wancata^3^, J. Simon^1^

Institutional affiliation of the authors:

^1^ Department of Health Economics, Center for Public Health, Medical University of Vienna, Kinderspitalgasse 15/I, 1090 Vienna, Austria; AL ORCID: 0000-0002-6689-3708, JS ORCID: [0000-0001-9279-8627](http://orcid.org/0000-0001-9279-8627)

^2^ Institute for Social Policy, Vienna University of Economics and Business, Welthandelsplatz 1, 1020 Vienna, [august.oesterle@wu.ac.at](mailto:august.oesterle@wu.ac.at); AÖ ORCID: 0000-0002-2213-8815

^3^ Clinical Division of Social Psychiatry, Department of Psychiatry and Psychotherapy, Medical University of Vienna, Währinger Gürtel 18-20, 1090 Vienna, [johannes.wancata@meduniwien.ac.at](mailto:johannes.wancata@meduniwien.ac.at); JW ORCID: 0000-0003-4951-6780

*Corresponding author:

Agata Łaszewska, Department of Health Economics, Center for Public Health, Medical University of Vienna, Kinderspitalgasse 15/I, 1090 Vienna, Austria, Tel: +43 1 40160–34844, E-mail: [agata.laszewska@meduniwien.ac.at](mailto:agata.laszewska@meduniwien.ac.at)

### **Table 1** Search strategy in electronic databases

|  | **Epidemiology** | **Mental Disorders** | **Austria** | **Database** |
| --- | --- | --- | --- | --- |
| **MeSH terms** | exp Epidemiology  exp Epidemiologic Studies  exp Prevalence  exp Incidence  exp Mortality  exp Morbidity | exp Mental Disorders  exp Mental Health  exp Mentally Ill Persons | exp Austria | MEDLINE |
| **EMTREE terms** | 'epidemiology'/exp  'prevalence'/exp  'incidence'/exp  'mortality'/exp  'morbidity'/exp | 'mental disease'/exp  'mental health'/exp  'mental patient'/exp | 'austria'/exp | EMBASE |
| **Free-text term** | prevalen*, incidence, mortality, epidemiolog* | “mental healt”, “psychiatric diseas*” , “psychiatric disorder*”, “psychiatric ill*”, (mental* adj3 disorder*), (mental* adj2 ill*), (mental adj2 disease*),  schizophren*, schizoaffective, unipolar, bipolar, depress*, anxiety, “personality disorder*”, “affective disorder*”, “stress disorder*”,”substance use”, “substance dependence”, (mood adj2 disorder*), , (eating adj2 disorder*), (delusional adj2 disorder*), (psychotic adj2 disorder*) | Austria*, Vienna, Salzburg, Tyrol, Styria, Carinthia, Vorarlberg, Burgenland, Graz, Innsbruck, Linz | MEDLINE, EMBASE, Scopus, PsycINFO, CINAHL, Social Science Citation Index (SSCI) and PSYNDEXplus. |

### **Table 2** Studies included in the review

| **Author and setting** | **Sample design** | **Sampling frame** | **Assessment** | **Disease area** | **Year of data** | **Sample size** | **Quality score and limitations** |
| --- | --- | --- | --- | --- | --- | --- | --- |
| **Prjek et al. 2016 [42]**  Austria – general population | Randomly selected phone numbers age: ≥18 | Austrian telephone books | Telephone interview | Seasonal affective disorder, major depression | Not reported | 910 | Score 6  Author reported limitations:   - study restricted to subjects with access to telephone - retrospective interviews which makes a memory bias possible - the sample not controlled for ethnicity which might be a further confounding factor - despite the random selection of subjects, some of the demographic properties of this sample were statistically significantly different compared to the general population |
| **Mangweth-Matzek et al. 2014 [54]**  Innsbruck – general population | Randomly selected female sample using census bureau data age: 40-60 | Census | Self-reported mailed questionnaires | Eating disorders | Not reported | 715 | Score 6  Author reported limitations:   - sent questionnaires – underestimation of eating disorders prevalence rates - self-reported information on diagnoses - low response rate |
| **Kerkenaar et al. 2013 [36]**  Austria – general population | Randomly selected phone numbers age: ≥15 | Random-digit dialing on a combined sample of mobile and landline phone numbers | Telephone interview | Depression and anxiety | 2010-2011 | 3,448 | Score 5  Author reported limitations:   - cross-sectional data which do not allow to determine casual relationships - PHQ-4 was used to identify depression cases instead of a structured psychiatric interview - GAD-2 and PHQ-2 are not culturally validated in countries of origin of migrant population in Austria and it is questionable whether the same cut-off values can be applied |
| **Von Dem Knesebeck et al. 2011 [55]**  Austria – general population | Randomly selected sample age: ≥25 | All private residents | Face-to-face interview + self-reported questionnaires | Depression | 2007 | 1,742 | Score 3  Author reported limitations:   - low response rate are lower in the highest and lowest socioeconomic groups and less healthy people - diagnostic measure (CES-D) based on self-report scale; respondents scoring high are not assumed to exhibit a clinical condition |
| **Kopp et al. 2011 [39]**  Austria – general population | Randomly selected sample age: ≥15 | All private residents | Face-to-face interview | People treated with psychotropic drugs | 2007 | 15,474 | Score 5  Author reported limitations:   - survey data are cross‐sectional and do not reveal the reason for treatment and lack of standardised diagnostic procedure concerning both diagnosis and severity of mental illness |
| **Mossaheb et al. 2009 [41]**  Vienna – general population | Inhabitants of the 21st and 22nd district of Vienna  age: ≥75 | Official voting registry | Face-to-face interview + self-reported questionnaires | Depression | 2005 | 331 | Score 5  Author reported limitations:   - personality traits were not extensively assessed - high number of refusal to participate in the study at baseline and follow-up - findings cannot be generalised to all elderly as they concern only population of 75-years-old individuals |
| **Castro-Costa et al. 2007 [35]**  Austria – general population | Households with at least one 50+ individual age: ≥50 | Telephone directories/International survey | Face-to-face interview + self-reported questionnaire | Depression | 2004 | 1,986 | Score 6  Author reported limitations:   - simple scale-based assessment used for depression instead of a clinical diagnostic interview |
| **Kapusta et al. 2006 [47]**  Lower Austria – general population | All males undergoing an examination for National Service in Lower Austria age: 18 | National Service Assessment | Self-reported questionnaires | Alcohol dependence | 2002 | 1,902 | Score 4-5  Author reported limitations:   - reduced intake of an illicit drugs prior the examination as the assessment dates where known to the study participants |
| **Mangweth-Matzek et al. 2006 [40]**  Innsbruck – general population | Randomly selected females age: 60 -70 | Census | Self-reported mailed questionnaires | Eating disorders | Not reported | 475 | Score 6  Author reported:   - high drop-out rate that limits the generalisability of findings - underestimation of the ED’s rates as the participants with ED are more often non-responders in prevalence studies than controls - using questionnaires for diagnosis instead of clinical interviews |
| **Weissgram et al. 2003 [45]**  Vienna – general population | Inhabitants of the 21. and 22. district of Vienna age: ≥75 | Official voting registry | Face-to-face interview | Depression | 2000 | 606 | Score 5  No author reported limitations |
| **Kinzl et al. 1999 [38]**  Tyrol – general population | Random selected sample of women from Tyrol age: 15-85 years | Census | Telephone interview | Eating disorders | 1997 | 1,000 | Score 4  Author reported limitations:   - information bias due to the self-report information |
| **Kinzl et al. 1999 [37]**  Tyrol – general population | Random selected sample of men from Tyrol age: 18-88 | Census | Telephone interview | Eating disorders | 1997 | 1,000 | Score 5  Author reported limitations:   - information bias due to the self-report information - reliability of phone interviews in case of BED when patients try to hide a disease |
| **Rathner & Dunkel 1998 [43]**  Austria – general population | Random sample | Telephone directories | Self-reported questionnaires | Alcohol dependence | 1996 | 498 | Score 6  Author reported limitations:   - self-report questionnaires |
| **Freidl et al. 2015 [48]**  Tyrol and Vienna, rural and urban areas – hospitals | Hospitals patients age: ≥18 | Patients admitted to hospital wards of two hospitals during the study period | Face-to-face interview | Psychiatric disorders in general hospital wards | Not reported | 821 | Score 4  Author reported limitations:   - small sample size for some diagnoses |
| **Kunz et al. 2014 [50]**  Salzburg - nursing homes and general hospitals | Nursing home residents and hospital patients age: >60 | Residents in 33 nursing homes and patients in 4 general hospitals | Face-to-face interview + self-administered questionnaires | Alcohol dependence | Not reported | 455 in nursing homes 123 inpatient | Score 3  No author reported limitations |
| **Stompe et al. 2010 [53]**  Vienna – forensic prison | Recruited prisoners age: ≥18 | Remand prisoners and convicted prisoners Justizanstalt Wien-Josefstadt | Face-to-face interview | Psychiatric disorders among prisoners | Not reported | 200 | Score 3  Author reported limitations:   - data are selective and provide only a general rough insight into the topic of mental health condition of remand prisoners and convicted prisoners in Austria - only one institution included in the study |
| **Mann et al. 2009 [51]**  Vorarlberg - nursing homes | Residents in nursing homes  age: | All residents of  nursing homes in Vorarlberg | Face-to-face interview | Psychotropic treatment | 2007 | 1,844 | Score 5  Author reported limitations:   - data on medication prescribed "as required" were not included - patients’ diagnoses not included - psychotropic medication use is not expected to be associated with mental health diagnoses |
| **Wancata et al. 1998 [52]**  Tyrol and Vienna – nursing homes | Residents in nursing homes  age: ≥18 | Residents admitted to nursing homes during the investigation period | Face-to-face interview | Psychiatric disorders among newly admitted nursing homes residents | 1991-1992 | 262 | Score 5  Author reported limitations:   - high prevalence of psychiatric disorders among admitted patients because elderly suffered from mental health problems have increased risk of admission to nursing homes |
| **Wancata et al. 1996 [49]**  Tyrol and Vienna - hospitals | Hospital patients  age: ≥18 | Patients admitted to hospital wards of two hospitals during the study period | Face-to-face interview | psychiatric disorders in general hospital wards | 1991-1992 | 784 | Score 5  No author reported limitations |

Note: BED - binge eating disorder; CES-D - Centre for Epidemiologic Studies Depression Scale; ED – eating disorders; GAD - Generalised Anxiety Disorder; PHQ - Patient Health Questionnaire

### **Table 3** Prevalence of different mental diseases in the general population

| **Disease** | **Period of prevlence** | **Prevalence [95% CI]** | | | **Men:women differences** | **Age range** | **Mean age^1^ (SD)** | **Instrument** | **Cut-off value** | **Diagnostic system** | **Instrument type** | **Ref.** |
| --- | --- | --- | --- | --- | --- | --- | --- | --- | --- | --- | --- | --- |
|  |  | **Total** | **Men** | **Women** |  |  |  |  |  |  |  |  |
| **Mood disorders** | | | | | | | | | | | | |
| Major depression | Ten-year | 15.60% [13.19–18.02] | 13.21% | 16.98% | No statistically significant difference | ≥18 | NR | SHQ | NA | DSM-5 | Screening instrument | [42] |
| Depression | Two-week | 9.9% | 10.8% | 9.1% | No statistically significant difference (p=0.098) | ≥15 | NR | PHQ-2 | ≥3 | NA | Screening instrument | [36] |
| Depression | One-week | NR | 12.4% | 13% | NR | ≥25 | NR | CES-D 8 | ≥10 | NA | Screening instrument | [55] |
| Depression | Point prevalence | 19.6% [17.8-21.3] | NR | NR | Higher prevalence of symptoms among women | 50-100 | NR | EURO-D | ≥4 | NA | Screening instrument | [35] |
| Depression (major, minor, subsyndromal) | Point prevalence | 16.3% (7.2%, 9.1%, 5.6%) | NR | NR | NR | ≥75 | NR | DSM-IV-based questionnaire | NA | DSM-IV | Structured interview | [45] |
| Dysphoric disorders (depression and anxiety) | Two-week | 6.7% | NR | NR | NR | ≥15 | NR | PHQ-4 (PHQ-2, GAD-2) | NA | NA | Screening instrument | [36] |
| Seasonal affective disorder | Ten-year | 2.53% [1.45-3.60] | 2.40% | 2.60% | No statistically significant difference | ≥18 | 56.8 (13.2) | SHQ | NA | DSM-5 | Screening instrument | [42] |
| **Anxiety disorders** | | | | | | | | | | | | |
| Anxiety | Two-week | 12% | 10% | 13.8% | Statistically significant difference (p<0.001) | ≥15 | NR | GAD-2 | ≥3 | NA | Screening instrument | [36] |
| **Eating disorders** | | | | | | | | | | | | |
| Eating disorders^2^ | Point prevalence | NA | NA | 4.6% [3.3–6.4%] | NA | 40-60 | NR | Questions adapted from SCID | NA | DSM-IV | Screening instrument | [54] |
| Eating disorders^2^ | Point prevalence | NA | NA | 3.6% [2.3–5.9%] | NA | 60-70 | NR | Questions adapted from SCID | NA | DSM-IV | Screening instrument | [40] |
| Binge eating disorder | Point prevalence | NA | 0.8% | 3.3% | NA | 15-88 | NR | Questions recommended by Bruce & Wilfley (1996) | NA | DSM-IV | Screening instrument | [37, 38] |
| Bulimia nervosa | Point prevalence | NA | 0.5% | 1.5% | NA | 18-88 | NR | Questions recommended by Bruce & Wilfley (1996) | NA | DSM-IV | Screening instrument | [37, 38] |
| **Alcohol dependence** | | | | | | | | | | | | |
| Alcohol dependence | Point prevalence | NA | 3.2% [2.4-4.0] | NA | NA | 18 | NR | CAGE | ≥2 | NA | Screening instrument | [47] |
| Alcohol dependence | Point prevalence | 2.2% [1.1-3.9] | NR | NR | The male: female ratio was 4:1 | ≥18 | NR | CAGE | 4 | NA | Screening instrument | [43] |
| Problematic drinking | Point prevalence | NA | 15.1% [13.5–16.8] | NA | NA | 18 | NR | CAGE | ≥1 | NA | Screening instrument | [47] |
| Problematic drinking | Point prevalence | 19.7% [16-24] | NR | NR | The male: female ratio was 3:1 | ≥18 | NR | CAGE | ≥2 | NA | Screening instrument | [43] |

Note: ^1^ Mean age of people affected by a disease; ^2^ Eating disorders defined as occurrence of symptoms meeting DSM-IV criteria for current anorexia nervosa, bulimia nervosa or EDNOS (Eating Disorder Not Otherwise Specified); NR – not reported; NA – not applicable; CES-D - Centre for Epidemiologic Studies Depression Scale; GAD - Generalised Anxiety Disorder; PHQ - Patient Health Questionnaire; SCID - Structured Clinical Interview; SHQ - Seasonal Health Questionnaire

### **Table 4** Prevalence of mental diseases in the institutionalised population

| **Disease & type of institution** | **Period of prevalence** | **Prevalence** | | | **Men:women differences** | **Age range** | **Mean age**^2^ **(SD)** | | **Instrument** | | **Cut-off value** | | **Diagnostic system** | | **Instrument type** | | **Ref.** |
| --- | --- | --- | --- | --- | --- | --- | --- | --- | --- | --- | --- | --- | --- | --- | --- | --- | --- |
|  |  | **Total** | **Men** | **Women** |  |  |  |  |  |  |  |  |  |  |  |  |  |
| **Mental diseases**^1^ | | | | | | | | | | | | | | | | | |
| Hospital | Point prevalence | 32.2 % | NR | NR | NR | ≥18 | NR | CIS | | NA | | DSM-III-R | | Clinical interview | | [48] | |
| Hospital | Point prevalence | 30.5% | 35% | 28.3% | No significant difference | ≥18 | NR | CIS | | NA | | ICD-9 | | Clinical interview | | [49] | |
| Nursing home | Point prevalence | 76.3% | 83.1% | 74.1% | Men suffered more  often from mental illness than women | ≥18 | NR | CIS | | NA | | ICD-9 | | Clinical interview | | [52] | |
| Prison | Point prevalence | RP: 69%  CP: 72% | NR | NR | NR | ≥18 | NR | SCAN | | NA | | ICD-10 | | Clinical interview | | [53] | |
| **Alcohol dependence** | | | | | | | | | | | | | | | | | |
| Hospital | Point prevalence | 0% | NA | NA | NR | >60 | NR | SCID | | NA | | DSM-IV | | Clinical interview | | [50] | |
| Hospital | Point prevalence | 10.6% | NR | NR | NR | >60 | NR | AUDIT | | ≥4 women, ≥5 men | | NA | | Screening instrument | | [50] | |
| Hospital | Point prevalence | 12.3% | 19% | 9% | Significantly higher prevalence among men and women | >60 | NR | SMAST-G | | ≥2 | |  | | Screening instrument | | [50] | |
| Nursing home | Point prevalence | 0.7% | NR | NR | NR | >60 | NR | SCID | | NA | | DSM-IV | | Clinical interview | | [50] | |
| Nursing home | Point prevalence | 5.7% | NR | NR | NR | >60 | NR | AUDIT | | ≥4 women, ≥5 men | | NA | | Screening instrument | | [50] | |
| Nursing home | Point prevalence | 5.5% | NR | NR | NR | >60 | NR | SMAST-G | | ≥2 | |  | | Screening instrument | | [50] | |
| **Substance abuse** | | | | | | | | | | | | | | | | | |
| Hospital | Point prevalence | 5.8% | NR | NR | NR | ≥18 | NR | CIS | | NA | | DSM-III-R | | Clinical interview | | [48] | |
| Hospital | Point prevalence | 8.1% | NR | NR | NR | ≥18 | NR | CIS | | NA | | ICD-9 | | Clinical interview | | [49] | |
| Nursing home | Point prevalence | 4.2% | NR | NR | NR | ≥18 | NR | CIS | | NA | | ICD-9 | | Clinical interview | | [52] | |
| Prison | Point prevalence | RP: 54%  CP: 57% | NR | NR | NR | ≥18 | NR | SCAN | | NA | | ICD-10 | | Clinical interview | | [53] | |
| **Depression** | | | | | | | | | | | | | | | | | |
| Hospital^3^ | Point prevalence | 7.9%, 2.1% | NR | NR | NR | ≥18 | NR | CIS | | NA | | DSM-III-R | | Clinical interview | | [48] | |
| Nursing home^4^ | Point prevalence | 5.7% | NR | NR | NR | ≥18 | NR | CIS | | NA | | ICD-9 | | Clinical interview | | [52] | |
| Prison^5^ | Point prevalence | RP: 13%  CP: 4% | NR | NR | NR | ≥18 | NR | SCAN | | NA | | ICD-10 | | Clinical interview | | [53] | |

Note: ^1^ Mental diseases of any kind; ^2^ Mean age of people affected by a disease; ^3^ Estimates of minor and major depression, respectively; ^4^ Estimates of depressive disorders defined according to ICD-9 as “depressive disorders not elsewhere classified” and “manic depressive disorders or other psychoses”; ^5^ Estimates of affective/mood disorders; NR – not reported; NA – not applicable; AUDIT - Alcohol Use Disorder Identification Test; CIS – Clinical Structured Interview; CP - convicted prisoners; RP - remand prisoners; SCAN - Schedules for Clinical Assessment in Neuropsychiatry; SCID - Structured Clinical Interview; SMAST-G - Short Michigan Alcoholism Screening Test
